# Nogo-A and NfL Levels in CSF from Newly Diagnosed Multiple Sclerosis and Neuromyelitis Optica Spectrum Disorder Patients Positive for Anti-HHV6-A IgG Autoantibody

**DOI:** 10.3390/jcm14155497

**Published:** 2025-08-05

**Authors:** Şeyda Karabörk, Bihter Gökçe Çelik, Firdevs Uluç, Şule Aydın Türkoğlu, Serpil Yıldız

**Affiliations:** 1 Department of Medical Microbiology, Faculty of Medicine, Bolu Abant İzzet Baysal University, Gölköy Campus Bolu, Bolu 14030, Türkiye; 2 Department of Interdisciplinary Neuroscience, Graduate School of Health Sciences Institute, Bolu Abant İzzet Baysal University, Bolu 14030, Türkiye; b.gokcebozat@hotmail.com (B.G.Ç.); frdvserim@gmail.com (F.U.); 3 Department of Neurology, Faculty of Medicine, Bolu Abant İzzet Baysal University, Bolu 14030, Türkiye; suleaydinturkoglu@hotmail.com (Ş.A.T.); serpilkuyucu@hotmail.com (S.Y.)

**Keywords:** demyelinating diseases, pseudotumour cerebri, human herpes virus, Nogo-A, neurofilament light chain

## Abstract

**Background:** Agents responsible for the initiation of autoimmune responses are still under investigation. The aim of this study was to determine Nogo-A and NfL levels in CSF samples from newly diagnosed multiple sclerosis (MS), neuromyelitis optica spectrum disorder (NMOSD) and pseudotumour cerebri (PTC) patients positive for HHV6-A IgG autoantibody. **Methods:** Initial CSF samples from 42 patients were analysed by ELISA. Independent samples t tests, Mann–Whitney U tests, crosstabulation with Fisher’s exact tests and Pearson/Spearman correlation analyses were used for group comparisons. **Results:** Anti-HHV6A IgG positivity was highest in MS, followed by NMOSD and then PTC (6.7%), but no significant difference in positivity was found among the groups (*p* = 0.367). No significant difference was found among the groups for NfL or Nogo-A levels (*p* = 0.373, *p* = 0.975, respectively). Anti-HHV6A negative MS cases had lower Nogo-A levels than positive cases (*p* = 0.046). In addition, anti-HHV6A negative PTC cases had lower Nogo-A levels than positive cases (*p* = 0.015). Anti-HHV6A positive MS patients had lower Nogo-A levels than the PTC positive group and this difference was very close to significant (*p* = 0.063). **Conclusions:** Anti-HHV6A positivity was found mainly in the MS group. Anti-HHV6A was found to be associated with Nogo-A levels, especially in the MS and PTC groups. Anti-HHV6A autoantibodies might play a role in the pathophysiology of MS.

## 1. Introduction

Multiple sclerosis (MS) is a T-cell mediated autoimmune disease in which the immune system attacks certain proteins in the myelin sheath of the central nervous system (CNS). This autoimmune response, which occurs at the onset of MS, is a process in which T-cell activation and migration play a primary role [1,2]. Although MS is known to be a T-cell mediated autoimmune disease, B cells also contribute significantly to the autoimmune response. The presence of OCB in the CSF, accumulation of antibodies and complement around the lesion and clonal expansion of B cells are the main conditions observed. In more aggressive cases, localization of B, T and plasma follicles in the subarachnoid space is observed. Drugs such as rituximab, ocrelizumab and ofatumumab are monoclonal antibody therapies directed against CD20, which consists of four transmembrane proteins on the surface of B cells [3,4].

NMOSD is an astrocytopathic disease of the CNS that can also cause inflammatory lesions in the midline of the brain and is dependent on anti-AQP4-associated B cells. B cells that produce antibodies against AQP4 are activated outside the CNS, and antibodies that pass through the disrupted BBB attack AQP4. Although it is not yet clear why and how the integrity of the BBB is disrupted, it is thought that it is disrupted after the induction of inflammation. In addition to B cells, T cells are also involved in the pathology of NMOSD. T cells induce inflammation in the CNS and cause the accumulation of neutrophils and eosinophils. It has been suggested that the accumulation of complement, one of the elements of the innate immune response, is associated with the loss of AQP4 and that AQP4 antibodies may be an activator of the complement system [5]. This stimulates inflammation and the formation of a membrane attack complex, leading to astrocytic damage. As a result, glial fibrillary acidic protein (GFAP) is released. In this case, because the target is AQP4, there is more astrocytic damage than myelin sheath or axonal damage, unlike in MS [6,7]. Damage to oligodendrocytes occurs with the induction of inflammation following axonal damage. Demyelination and subsequent axonal damage and neuronal death occur because of damage to the oligodendrocytes that contribute to the production of the myelin sheath [8].

Pseudotumour cerebri (PTC) is defined as an increase in intracranial pressure. Increased cerebrospinal fluid (CSF) content and increased cerebral venous circulation lead to increased intracranial pressure. The mechanism causing the imbalance in CSF content is still under investigation. PTC is divided into two groups: primary PTC with no identifiable cause and secondary PTC with an identifiable secondary cause. Secondary causes include thrombosis, paranasal sinus abnormalities, anaemia, excessive vitamin A intake, polycystic ovary syndrome and Addison’s disease [9]. Pseudotumoral lesions can usually be diagnosed using imaging techniques without the need for biopsy. These lesions are most associated with MS and, to a lesser extent, NMOSD or acute disseminated encephalomyelitis (ADEM). Patients with pseudotumoral lesions have a favourable prognosis, but close follow-up is necessary [10].

The role of viruses in the aetiology of MS is of great interest as reflected in the data from studies conducted over many years [11,12,13]. HHV6 is a member of the Roseolovirus genus of the beta herpes virus family, which contains double-stranded DNA, and is one of the viruses thought to be involved in the aetiology of MS [14]. There are two known variants of HHV6, HHV6A and HHV6B [15]. There are only a few protein differences between these two variants. HHV6A exerts its effect by binding to the CD46 receptor. In humans, this receptor is present on all nucleated cells. HHV6B can also use CD46 for infection but primarily uses the CD134 receptor [15].

The presence of high levels of HHV6A viral genes and the expression of mRNA and protein in oligodendrocytes and CSF samples from MS patients has been demonstrated, and it has been suggested that MS and HHV6A infection may be associated [16]. It has been reported that the presence of HHV6A antibodies increases the risk of conversion from CIS (clinically isolated syndrome) to MS and that HHV6 antibody levels are higher during MS relapses. Higher disability scores are associated with higher HHV6 antibody levels [17]. It has been reported that high HHV6A antibody titres during MS relapses are reduced by natalizumab treatment, and HHV6A may be an early biomarker of drug response [11,18]. It is emphasized that more research is needed to better understand the relationship between HHV6 and MS [19].

Neurofilament light chains (NfL) are cylindrical proteins found in the cytoplasm of nerve cells. They are released in small amounts under normal physiological conditions. It is known that the amount of NfL released increases 2.5-fold, particularly after the age of 50, and this rate increases exponentially with age. Higher levels of NfL are released into the extracellular fluid, cerebrospinal fluid and blood, particularly in neurodegenerative, inflammatory, vascular and traumatic diseases where neuroaxonal damage occurs [20,21]. NfL has been reported to be an important candidate biomarker in diseases such as MS [22], NMOSD [23], Alzheimer’s disease, frontotemporal dementia, amyotrophic lateral sclerosis, Huntington’s disease, atypical Parkinson’s disease and traumatic brain injury [24,25]. MS and NMOSD patients have been reported to have higher serum NfL levels than healthy subjects [23]. The fact that NfL is a non-specific marker of axonal damage suggests that it may not be able to discriminate between neurological diseases with similar levels of axonal damage. It is thought that evaluation of NfL in combination with various neuronal autoantibodies or herpesvirus positivity may help to make this distinction [26].

Neurite growth inhibitor-A (Nogo-A) is a protein that belongs to the reticulon family of mostly membrane-associated proteins and is encoded by the RTN4 gene. Nogo-A, the largest member of the reticulon family, consists of 1192 amino acids. It was first characterized in 1988 as an inhibitor of neurite outgrowth in brain and spinal cord myelin in vitro [27]. Nogo-A is expressed by oligodendrocytes, mainly in the endoplasmic reticulum and to a lesser extent on the cell surface of the myelin sheath surrounding the axon. Some recovery can occur after tissue injury, which may be explained by neuronal plasticity after injury. It is thought that activation of astrocytes and microglia after tissue damage may create an unfavourable environment for axonal regeneration, and inhibitory proteins such as Nogo-A inhibit neuronal plasticity. It is known that when neurons are damaged, blocking Nogo-A protects the damaged tissue. NgR1, to which Nogo-A binds, is associated with MAG, MOG and chondroitin sulphate proteoglycans [28,29].

In experimental models of neuroinflammatory diseases, including MS and spinal cord injury, Nogo-A has been suggested to play a regulatory role and may be a potential therapeutic target in MS [30]. In addition to NgR1 being a therapeutic target for MS, neurotropic viruses are known to bind this receptor to spread throughout the CNS. Neurotropic viruses bind to NgR1 together with an associated molecule called JAM-A (Junctional Adhesion Molecule-A) to spread haematogenously [31,32]. Given the important role of the NgR1-bound JAM-A molecule in cell biology, it is emphasized that it forms junctions between AQP4 and cell membranes and therefore its relationship with aquaporins should be investigated [33].

## 2. Materials and Methods

### 2.1. Study Design and Population

Ethical approval for our study was obtained from Bolu Abant İzzet Baysal University (BAIBU) Clinical Research Ethics Committee on 4 February 2020, decision number 2020/16, and was supported by BAIBU Scientific Research Projects Unit Grant No. 2021.08.32.1503.

A total of 42 people with a pre-diagnosis of MS and other demyelinating diseases who were admitted to the outpatient neurology department of the BAIBU Training and Research Hospital between March 2020 and January 2022 and who agreed to participate by completing an informed consent form were included in this study. As a lumbar puncture (LP) could not be performed in healthy subjects, the control group (unhealthy control) consisted of patients diagnosed with PTC, for which there are few publications reporting inflammation. After obtaining the necessary informed consent from the patients, the CSF samples (3–5 mL) obtained by the LP method were stored at −80 °C until the time of the study.

MS cases were determined according to the 2017 revised McDonald criteria, NMOSD cases according to the 2015 International NMO Diagnostic Panel criteria, and PTC cases according to the modified Dandy criteria [34]. All MS cases were selected as the RRMS subtype. NMOSD cases consisted of ON and TM subtypes. PTC cases consisted of the primary subtype, i.e., of unknown cause. A total of 42 cases were included, n = 19 (16 female, 3 male) for the MS group, n = 8 (5 female, 3 male) for the NMOSD group and n = 15 (13 female, 2 male) for the PTC group. The levels of anti-HHV6A IgG autoantibodies, NfL and Nogo-A in the cases (MS and NMOSD) and controls (PTC) were determined by ELISA (Sino-GeneClon Biotech Co., Ltd., HangZhou, China) according to the manufacturer’s recommendations.

### 2.2. Statistical Analysis

Data were analysed using the statistical package SPSS Windows 22.0. The 95% confidence level was used. Frequencies (n) and percentages (%) were used for categorical variables and mean, standard deviation (SD) and median for numerical variables. Frequency tables and the exact method of Fisher’s chi-squared test [35] were used to compare independent categorical variables such as gender, age at onset, OCD types, EDSS scores and anti-HHV6A positivity. For normally distributed numerical variables such as age and Nogo-A, the one-way analysis of variance (ANOVA)–Bonferroni test was used to compare multiple groups, and the independent samples t-test was used to compare paired groups. For non-normally distributed numerical values such as IgG index and NfL, the Kruskal–Wallis test was used to compare multiple groups and the Mann–Whitney U test was used to compare paired groups. Pearson correlation analysis was used for normally distributed parameters and Spearman correlation analysis for non-normally distributed data to determine the relationships among all parameters, and *p* < 0.05 was considered statistically significant.

## 3. Results

When the normally distributed age data obtained from all subjects were analysed by one-way analysis of variance, no significant difference was found among the mean ages of the three groups (*p* = 0.259). Early-onset cases were more common in the MS group, whereas late-onset cases were more common in the PTC group. There was no significant difference in the percentage of early-onset and late-onset cases when comparing within and between groups (*p* = 0.363). The majority of the 44 patients (16 female, 3 male for MS; 5 female, 3 male for NMOSD; 13 female, 2 male) in our study were female (77.3%). After analysing our data and considering the gender differences in the sample, it appears that the important outcomes of Nogo-A, IgG index and NfL levels were not significantly affected by gender. Although there was no significant difference between the proportions of women and men in the three groups, the proportion of women was higher (*p* = 0.221). When comparing OCB types, as expected, type 2 negativity was observed at the highest rate in MS patients, type 1 and type 2 negativity were observed equally in the NMOSD group, and type 1 negativity was observed at the highest rate in the PTC group (*p* = 0.001). No significant difference was found when other OCB types were compared between groups. When the IgG indices of the subjects in the groups were compared, it was observed that the MS group had higher IgG levels than both the NMOSD (*p* = 0.001) and PTC (*p* = 0.015) groups, and the NMOSD group had higher IgG index levels than the PTC group (*p* = 0.032). When comparing the EDSS scores of the patients, the frequency of score 2 was higher in the MS group and the frequencies of scores 3 and 7 was lower in the NMOSD group (*p* = 0.001). EDSS scoring was not performed in the PTC group.

**Figure 1:**
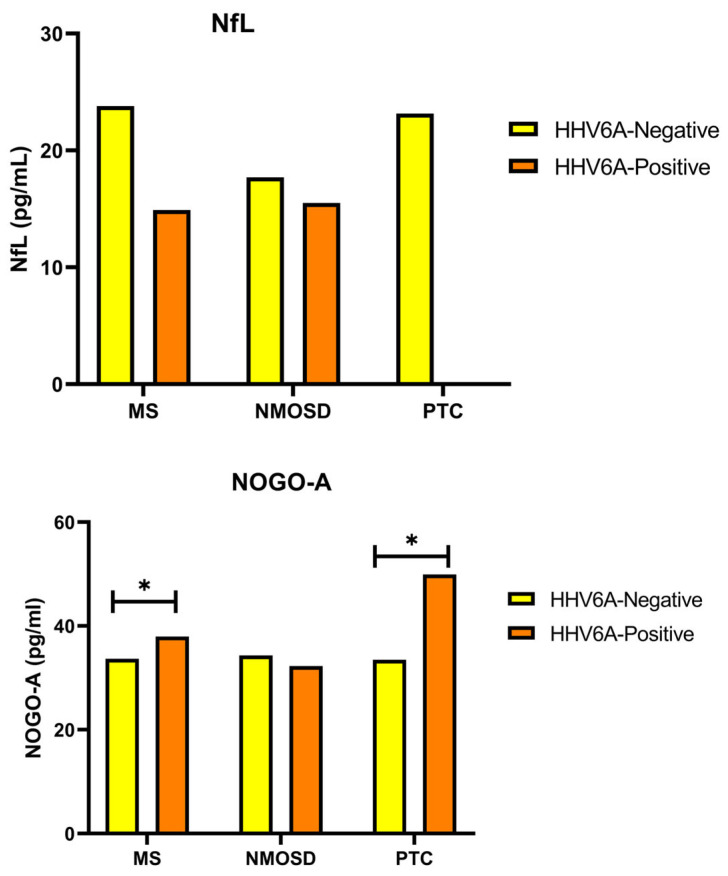
The bar graphs represent the NfL and Nogo-A status of the case (MS and NMOSD) and control (PTC) groups. Kruskal–Wallis test was used to compare the groups. HHV6A: anti-human herpes virus type 6A; Nogo-A: neuritis growth inhibitor-A; MS: multiple sclerosis; NMOSD: neuromyelitis optica spectrum disorder; PTC: pseudotumour cerebri; *p* < 0.05. * A trend toward significance was observed between HHV6A-positive MS and PTC groups (*p* = 0.063), indicating a potential difference in Nogo-A levels.

When analysing the anti-HHV6A IgG positivity of the patients in all groups, although the percentages of positivity were similar in all three groups, the highest percentage of positivity was in the MS group and there was no significant difference in positivity among the groups (*p* = 0.367) (Table 1), (Figure 1).

When NfL and Nogo-A levels were compared in all groups, no significant difference was found among the groups (*p* = 0.373 and *p* = 0.975, respectively) (Table 2), (Figure 1).

When comparing Nogo-A levels between the anti-HHV6A IgG autoantibody negative/positive MS, NMOSD and PTC groups, Nogo-A levels were lower in HHV6A negative MS cases than in positive MS cases (*p* = 0.046). In addition, Nogo-A levels were lower in HHV6A negative PTC cases than in positive PTC cases (*p* = 0.015). When comparing HHV6A positive MS and PTC cases, the Nogo-A level of the MS group was lower than that of the PTC group and this difference was very close to significance (*p* = 0.063) (Figure 1).

**Figure 2:**
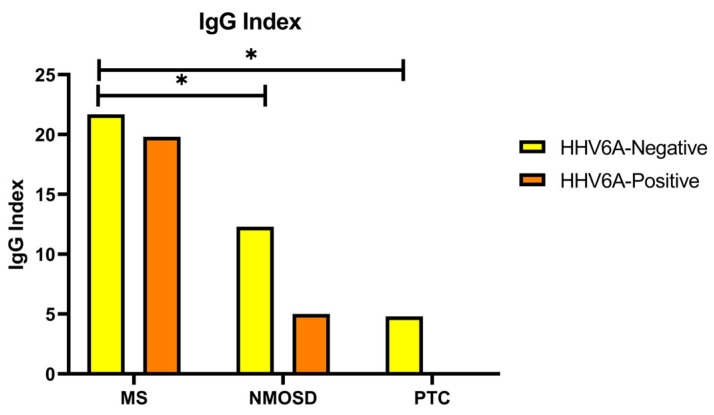
The bar graphs represent the IgG index according to the HHV6A negative/positive status of the cases. Kruskal–Wallis test was used to compare the groups. HHV6A, anti-human herpes virus type 6A; Nogo-A: neuritis growth inhibitor-A; MS, multiple sclerosis; NMOSD, neuromyelitis optica spectrum disorder; PTC, pseudotumour cerebri; *p* < 0.05. The asterisk represents statistical significance for the IgG index level of anti-HHV6A IgG autoantibody negative/positive patients among groups.

When the IgG index level of anti-HHV6A IgG autoantibody negative/positive patients in the MS, NMOSD and PTC groups were compared, IgG index levels of anti-HHV6A IgG negative MS patients were higher than those of anti-HHV6A IgG negative NMOSD patients (*p* = 0.019) and PTC patients (*p* = 0.002). The IgG index values of NMOSD patients who were anti-HHV6A IgG negative were higher than those of PTC patients who were anti-HHV6A IgG negative, and this difference was very close to significant (*p* = 0.061) (Figure 2).

When the relationship between parametrically distributed biomarkers of MS cases was evaluated by Pearson correlation analysis, a moderate positive correlation was found between age and OCB positivity (*p* = 0.044). There was a moderate positive correlation between age at disease onset and OCB positivity (*p* = 0.025). There was a moderate positive correlation between EDSS score and HHV6A positivity (*p* = 0.042). There was a moderate positive correlation between Nogo-A levels and HHV6A positivity (*p* = 0.046) (Table 3).

When the relationship between parametric distributed biomarkers of NMOSD cases was evaluated by Pearson correlation analysis, a strong positive correlation was found between OCB positivity and HHV6A positivity (*p* = 0.008) (Table 3).

When the relationship between parametrically distributed biomarkers of PTC cases was evaluated, a very strong and positive correlation was found between mean age and OCB positivity (*p* = 0.014). A strong and positive correlation was found between Nogo-A levels and anti-HHV6A positivity (*p* = 0.011) (Table 3).

When the relationship between non-parametrically distributed biomarkers of NMOSD cases was evaluated by Spearman correlation analysis, a strong and positive correlation was found between age at disease onset and NfL levels (*p* = 0.027). A very strong and positive correlation was found between mean age and NfL levels (*p* = 0.004). A strong and negative correlation was found between EDSS scores and IgG indices of the cases (*p* = 0.042). When the relationship between non-parametrically distributed biomarkers of PTC patients was evaluated by Spearman correlation analysis, a moderate positive correlation was found between Nogo-A levels and NfL levels (*p* = 0.031) (Table 4).

## 4. Discussion

In this study, demographic and biomarker data of patients in the MS, NMOSD and PTC groups were compared. Significant differences were observed between OCB types, and it was found that the rate of type 2 negativity was higher in MS patients. When IgG indices were analysed, the MS group had higher IgG levels than both the NMOSD and PTC groups. Nogo-A levels were significantly lower in HHV6A negative MS cases than in positive MS cases. A similar result was observed in PTC cases, and it was found that HHV6A negative PTC cases had lower Nogo-A levels than positive PTC cases. NfL levels were found to be positively correlated with age, and there was a similar positive correlation with age of disease onset.

At the diagnostic stage, the differential diagnosis of MS and NMOSD is very difficult. New biomarkers are needed to definitively differentiate these two diseases. Therefore, in this study, ELISA was used to detect anti-HHV6A IgG autoantibody positivity and Nogo-A and NfL levels in CSF samples from newly diagnosed MS and NMOSD cases in the relapsing phase. Patients diagnosed with PTC (unhealthy control) were studied as a control group.

MS and NMOSD trials are usually conducted in patients who have received treatment. Especially in drug-treated patients, some biomarkers may become negative, and the reliability of the results obtained is reduced [26]. In our study, the use of CSF samples from newly diagnosed cases made it possible to rule out any drug effect. Providing information on the changes that occur in the early stages of the disease enhances the quality of the results obtained in this study. When the demographic characteristics of the cases were evaluated, it was observed that the female population was larger than the male population, in accordance with the literature [36]. When the mean age of the groups was analysed, it was found that the mean age of the women was 34, while the mean age of the men was 31. It was found that there was no significant difference between the sexes or between the groups. It was important that there was no significant difference between the mean ages, both in terms of disease prognosis and the fact that some of the biomarkers studied are not affected by age. When all participants were grouped according to age at disease onset, about 57% were early-onset and 43% late-onset. When OCB positivity and IgG indices were analysed, type 2 positivity was, as expected, mostly observed in MS patients. The percentage of type 3 and type 4 OCB was higher in patients with late onset compared to those with early onset.

The presence of different OCB patterns in the CSF of PTC patients is well known. The presence of these OCB is associated with visual loss [37] and is reported to indicate the presence of an ongoing inflammatory process [38]. In our study, it was observed that 60% of PTC patients had no CSF OCB or IgG index values. When these patients were analysed, anti-HHV6A positivity, although not significant, was observed at a lower level. It is suggested that the reason for the lack of need for clinic referral may be related to the low positivity.

The pathology of NMOSD is complex. As a result, it is difficult to determine the diagnosis and treatment regimen. For this reason, biomarker studies are very important. One of the most important markers distinguishing MS from NMOSD is the IgG index level. This biomarker, which indicates impaired BBB integrity, is higher in MS patients [39]. In our study, the OCB and IgG index were not requested in one patient each from MS and NMOSD patients and in nine PTC patients. We believe that this may be because PTC patients are known to be clinically benign.

When all participants in our study were evaluated, the percentage of patients with an EDSS score of two was the highest. While a score of two was the highest in the MS group, scores of three and seven were more common in the NMOSD group. The disease processes in NMOSD patients are more severe than in MS patients. It has been shown that there is a strong positive correlation with higher EDSS scores in subjects with high anti-HHV6A IgG titres [17]. Another study showed that anti-HHV-6 seropositive MS cases had higher EDSS scores than seronegative cases [40]. Another study showing that EDSS scores of anti-HHV-6 seropositive MS cases were higher than seronegative cases, which emphasized that anti-HHV-6 plays an important role in the pathogenesis of MS [41]. Consistent with the literature, a positive correlation was found between EDSS score and anti-HHV6A positivity in MS patients in our study. This suggests that anti-HHV6A positivity affects the prognosis of the disease.

HHV6A is one of the viral agents implicated in the aetiology of MS. When evaluated on a group basis, anti-HHV6A IgG positivity was found to be highest in MS (26.3%), followed by NMOSD (12.5%) and lowest in PTC (6.7%). A 2022 study reported important findings on how HHV6 positivity increases the risk of MS. The presence of KIR2DL2 expression has been reported to increase the susceptibility of MS patients to HHV infection. In addition, a higher viral load was observed in MS patients compared to controls, and the percentage of cases with IgG positive for HHV6 was reported to increase in KIR2DL2-positive MS patients [42]. HHV-6A and HHV-6B are closely related to human herpesviruses, but studies suggest that HHV-6A, rather than HHV-6B, is associated with demyelinating diseases such as MS [17,19]. HHV-6A has been shown to increase the expression of genes associated with oligodendrocyte function and may affect Nogo-A levels by altering myelin and neuron-associated proteins. In contrast, there is limited data on the effect of HHV-6B on Nogo-A. Further studies are needed to determine whether HHV-6B affects Nogo-A levels in the same way as HHV-6A or in a different way. Regarding IgG index, both HHV-6A and HHV-6B may correlate with IgG antibody responses but may not affect CSF IgG index levels in the same way. Given its stronger association with neuroinflammatory responses, HHV-6A may have a more pronounced effect on IgG index levels due to its involvement in chronic immune activation. HHV-6B, which is usually associated with roseola cases and less with chronic neuroinflammation, is not expected to significantly alter the IgG index in neuroinflammatory contexts such as MS. As for NfL, although both HHV-6A and HHV-6B can cross the blood–brain barrier, the association of HHV-6A with neurodegenerative and inflammatory processes suggests that it may have a stronger effect on NfL levels than HHV-6B. As HHV-6B is less associated with persistent neuroinflammation, it is predicted that it will not increase NfL levels to the same extent [19,43,44,45].

Almost all patients in whom OCB is not requested for PTC in the clinic are HHV6A negative. One study examining HHV6 reactive oligoclonal bands in MS emphasized that the presence of herpes virus reactive OCBs in CSF further strengthens the association of MS with these viruses. It is also suggested that herpes viruses may be associated with the appearance of active lesions and should be investigated for new therapeutic strategies to treat these viruses in MS [46].

It has been reported that Nogo-A expression in MS brain tissue is increased in lesions close to oligodendrocytes and that this increase may play a role in demyelination [47]. Consistent with the literature, in our results, Nogo-A levels were higher in HHV6A-positive MS and PTC cases than in negative cases. In addition, Nogo-A levels were higher in HHV6A-positive PTC cases than in MS cases. Furthermore, HHV6A positivity was associated with Nogo-A levels in both MS and PTC cases. In this case, the relationship between HHV6A and Nogo-A in the differential diagnosis of MS and NMOSD may be a new research topic. In addition, it is thought that the higher level of Nogo-A in PTC cases compared to NMOSD cases may reflect the risk of conversion of PTC cases to MS or NMOSD cases in the future. In future studies, with new studies showing myelin damage in the presence of HHV6 in PTC cases, clearer information about the prognosis of the disease can be obtained.

It has been reported that the presence of NfL is observed during the diagnostic phase of the disease [48,49] and at disease onset [46]. In our study, the use of CSF samples obtained from newly diagnosed cases reflects the level of NfL at the stage of diagnosis and at disease onset. In our study, the NfL level was found to be positively correlated with age at disease onset. The faster progression of disability in people with late onset of the disease may be related to increased levels of NfL. It was concluded that the amount of Nogo-A increases as the amount of NfL increases, especially in PTC cases. This situation shows the severity of the pathology in PTC cases.

Nogo-A acts as an inhibitor of neuronal growth in myelin. Expression of Nogo-A and its receptors has been reported in lesional areas of patients with MS [47]. It is known to inhibit axon regeneration after axonal injury and to arrest neuronal growth and plasticity [50,51]. In our study, the Nogo-A levels of the MS NMOSD and PTC groups were very close to each other. In this case, we obtained more significant results when we compared the subjects in the groups according to their viral positivity/negativity status. According to these results, it was concluded that HHV6A positivity is associated with Nogo-A levels in MS and PTC cases.

Our study also analysed the effect of the gender distribution in the sample on the levels of Nogo-A, IgG index and NfL, and found that the results did not differ significantly according to gender. This finding suggests that gender may not have a direct effect on the levels of these biomarkers.

Nogo-A, IgG index and NfL levels are important biomarkers reflecting CNS damage, inflammation and neurodegenerative processes. Although it has been reported in the literature that the levels of some biomarkers vary according to gender, no such effect on specific biomarkers was observed in this study. This may suggest that CNS-derived biomarkers such as Nogo-A and NfL may be less sensitive to a specific sex hormone or to sex-related physiological differences.

The limited effect of sex on the biomarkers supports that these biomarkers can be used as reliable indicators to measure neurological damage and inflammation regardless of sex. Such results may be particularly valuable in clinical practice and in situations where more information is needed on whether sex influences biomarker measurement.

The important limitation of this study is that we have limited data for statistical calculations for some parameters, because the number of cases in the disease groups is low, due to the low number of newly diagnosed cases (no treatment process has been initiated), taken from cases presenting at the clinic of a peripheral hospital in the province of Bolu. Another limitation is the lack of a healthy control group due to the inability to obtain CSF samples from healthy individuals compared to studies conducted on serum samples. The PTC cases included in this study as controls had a pathology like that of MS and NMOSD cases. We believe that the investigation and follow-up of PTC cases, which are known to be benign, should be carried out in more detail.

## 5. Conclusions

Anti-HHV6A positivity was found mainly in the MS group. Anti-HHV6A was found to be associated with Nogo-A levels, especially in the MS and PTC groups. Anti-HHV6A autoantibodies might play a role in the pathophysiology of MS. HHV6A seems to be very effective in the differential diagnosis of MS, even if its positivity is low. The relationship between HHV6A and Nogo-A may provide a new mechanism in the differential diagnosis of MS and NMOSD. HHV6A seropositivity and its possible relationship with the other biomarkers that cause axonal damage could be differential for the diagnosis of MS and PTC cases.

In addition, the results of Nogo-A and NfL provide important insights into the pathophysiology of MS and PTC. The association of Nogo-A with HHV6A positivity highlights the role of the immune system in disease processes, while the association of NfL with age and disease severity demonstrates the potential of these biomarkers in clinical applications. We believe that these findings provide an important basis for future studies with large sample sizes and warrant further investigation into how these biomarkers can be used in clinical management and diagnosis.

## Figures and Tables

**Table 1:** Anti-HHV6A IgG positivity of cases in disease groups.

			MS	NMOSD	PTC
HHV6A	Negative	Number of cases (n)	14	7	14
		Percentage (%)	73.7	87.5	93.3
	Positive	Number of cases (n)	5	1	1
		Percentage (%)	26.3	12.5	6.7
*p*			0.367 ^f^		

^f^: Crosstabulation Fisher’s exact test, *p* ˂ 0.05, significant difference; *p* ˃ 0.05, no significant difference. MS, multiple sclerosis; NMOSD, neuromyelitis optica spectrum disorder; PTC, pseudotumour cerebri; HHV6A, anti-human herpes virus type 6A IgG autoantibodies.

**Table 2:** NfL and Nogo-A levels in case (MS and NMOSD) and control (PTC) groups.

		n	Median	Mean	STD	Min.	Max.	*p*
NfL	MS	19	102.00	110.16	25.08	76.00	169.00	0.373 ^k^
	NMOSD	8	101.82	94.08	23.45	42.00	116.00	
	PTC	15	105.00	107.07	13.45	74.00	124.00	
Nogo-A	MS	19	34.37	34.81	4.16	28.05	45.16	0.975 ^a^
	NMOSD	8	34.50	34.60	7.50	23.84	43.84	
	PTC	15	34.06	34.35	6.79	24.11	49.90	

^k^: Kruskal–Wallis test; ^a^: one-way analysis of variance; *p* ˂ 0.05, significant difference; *p* ˃ 0.05, not significant difference. MS, multiple sclerosis; NMOSD, neuromyelitis optica spectrum disorder; PTC, pseudotumour cerebri; NfL, neurofilament light chain; Nogo-A, neurite growth inhibitor-A; SD, standard deviation; Min, minimum value; Max, maximum value.

**Table 3:** Pearson correlation analysis results for MS, NMOSD and PTC cases.

MS		Age of Disease Onset	Age	OCB	EDSS	HHV6A	Nogo-A
Age of disease onset	r	1					
	*p*						
Age	r	0.874 **	1				
	*p*	0.000					
OCB	r	0.526 *	0.480 *	1			
	*p*	0.025	0.044				
EDSS	r	0.198	0.077	0.112	1		
	*p*	0.431	0.760	0.669			
HHV6A	r	0.108	0.016	−0.263	0.483 *	1	
	*p*	0.659	0.948	0.291	0.042		
Nogo-A	r	0.360	0.147	0.241	0.230	0.463 *	1
	*p*	0.130	0.549	0.335	0.358	0.046	
**NMOSD**		**Age of Disease Onset**	**Age**	**OCB**	**EDSS**	**HHV6A**	**Nogo-A**
HHV6A	r			0.884 **			
	*p*			0.008			
**PTC**		**Age of Disease Onset**	**Age**	**OCB**	**EDSS**	**HHV6A**	**Nogo-A**
The average age	r	0.812 **					
	*p*	0.000					
OCB	r		0.902 *				
	*p*		0.014				
Nogo-A	r					0.633 *	
	*p*					0.011	

r: Pearson’s correlation coefficient; *: *p* ˂ 0.05, **: *p* ˂ 0.01. r: 0–0.3/weak; 0.3–0.6/moderate; 0.6–0.8/strong; 0.8–1.0/very strong. OCB, oligoclonal band; EDSS, Expanded Disability Status Scale; HHV6A, anti-human herpes virus type 6A IgG antibody; Nogo-A, neuritis growth inhibitor-A.

**Table 4:** Spearman correlation analysis results for NMOSD and PTC cases.

NMOSD		IgG Index	NfL
Age of disease onset	r	0.144	0.764 *
	*p*	0.758	0.027
Age	r	0.464	0.881 **
	*p*	0.294	0.004
OCB	r	0.116	0.694
	*p*	0.805	0.083
EDSS	r	−0.771 *	−0.420
	*p*	0.042	0.300
HHV6A	r	−0.612	−0.082
	*p*	0.144	0.846
NfL	r	0.714	1.000
	*p*	0.071	
Nogo-A	r	−0.286	−0.095
	*p*	0.535	0.823
**PTC**		**IgG Index**	**NfL**
Nogo-A	r		0.558 *
	*p*		0.031

r: Spearman’s correlation coefficient, *: *p* ˂ 0.05; **: *p* ˂ 0.01. r: 0–0.3/weak; 0.3–0.6/moderate; 0.6–0.8/strong; 0.8–1.0/very strong. HHV6A, anti-human herpes virus type 6A IgG antibody; NfL, neurofilament light chain; Nogo-A, neurite growth inhibitor-A; IgG index, immunoglobulin G index; MS, multiple sclerosis; OCB, oligoclonal band; EDSS, Expanded Disability Status Scale.

## Data Availability

The datasets used in this study are available from the corresponding author upon reasonable request.
